# Fibromyalgia Is Correlated with Retinal Nerve Fiber Layer Thinning

**DOI:** 10.1371/journal.pone.0161574

**Published:** 2016-09-01

**Authors:** Elena Garcia-Martin, Javier Garcia-Campayo, Marta Puebla-Guedea, Francisco J. Ascaso, Miguel Roca, Fernando Gutierrez-Ruiz, Elisa Vilades, Vicente Polo, Jose M. Larrosa, Luis E. Pablo, Maria Satue

**Affiliations:** 1 Ophthalmology Department, Miguel Servet University Hospital, Zaragoza, Spain; 2 Aragones Institute of Health Research, IIS-Aragon, Zaragoza, Spain; 3 Psychiatry Department, Red de Investigación en Atención primaria (REDIAPP), Miguel Servet University Hospital, Zaragoza, Spain; 4 Ophthalmology Department, Lozano Blesa University Hospital, Zaragoza, Spain; 5 Institut Universitari d'Investigació en Ciències de la Salut (IUNICS), University of Balearic Islands, Palma de Mallorca, Spain; Massachusetts Eye & Ear Infirmary, Harvard Medical School, UNITED STATES

## Abstract

**Objective:**

To investigate whether fibromyalgia induces axonal damage in the optic nerve that can be detected using optical coherence tomography (OCT), as the retinal nerve fiber layer (RNFL) is atrophied in patients with fibromyalgia compared with controls.

**Methods:**

Fibromyalgia patients (n = 116) and age-matched healthy controls (n = 144) were included in this observational and prospective cohort study. All subjects underwent visual acuity measurement and structural analysis of the RNFL using two OCT devices (Cirrus and Spectralis). Fibromyalgia patients were evaluated according to Giesecke’s fibromyalgia subgroups, the Fibromyalgia Impact Questionnaire (FIQ), and the European Quality of Life-5 Dimensions (EQ5D) scale. We compared the differences between fibromyalgia patients and controls, and analyzed the correlations between OCT measurements, disease duration, fibromyalgia subgroups, severity, and quality of life. The impact on quality of life in fibromyalgia subgroups and in patients with different disease severity was also analyzed.

**Results:**

A significant decrease in the RNFL was detected in fibromyalgia patients compared with controls using the two OCT devices: Cirrus OCT ganglion cell layer analysis registered a significant decrease in the minimum thickness of the inner plexiform layer (74.99±16.63 vs 79.36±3.38 μm, respectively; p = 0.023), nasal inferior, temporal inferior and temporal superior sectors (p = 0.040; 0.011 and 0.046 respectively). The Glaucoma application of the Spectralis OCT revealed thinning in the nasal, temporal inferior and temporal superior sectors (p = 0.009, 0.006, and 0.002 respectively) of fibromyalgia patients and the Axonal application in all sectors, except the nasal superior and temporal sectors. The odds ratio (OR) to estimate the size effect of FM in RNFL thickness was 1.39. RNFL atrophy was detected in patients with FIQ scores <60 (patients in early disease stages) compared with controls in the temporal inferior sector (78.74±17.75 vs 81.65±3.61; p = 0.020) and the temporal superior sector (78.20±14.50 vs 80.74±3.88; p = 0.039) with Cirrus OCT; in the temporal inferior sector (145.85±24.32 vs 150.18±19.71; p = 0.012) and temporal superior sector (131.54±20.53 vs 138.13±16.67; p = 0.002) with the Glaucoma application of the Spectralis OCT; and in all sectors, except the average, nasal superior, and temporal sectors, and parameters with the Axonal application of the Spectralis OCT. Temporal inferior RNFL thickness was significantly reduced in patients with severe fibromyalgia (FIQ≥60) compared with patients with mild fibromyalgia (FIQ<60; 145.85±24.32 vs 138.99±18.09 μm, respectively; 145.43±13.21 vs 139.85±13.09 μm, p = 0.032 with the Glaucoma application and p = 0.021 with the Axonal application). The subgroup with biologic fibromyalgia exhibited significant thinning in the temporal inferior and superior sectors (115.17±20.82 μm and 117.05±24.19 μm, respectively) compared with the depressive (130.83±22.97 μm and 127.71±26.10 μm, respectively) and atypical (128.60±26.54 μm and 125.55±23.65 μm, respectively) subgroups (p = 0.005 and 0.001 respectively).

**Conclusions:**

Fibromyalgia causes subclinical axonal damage in the RNFL that can be detected using innocuous and non-invasive OCT, even in the early disease stages. The impact on the RNFL in the temporal sectors is greater in patients with biologic fibromyalgia, suggesting the presence of neurodegenerative processes in this subgroup of patients with fibromyalgia.

## Introduction

Fibromyalgia (FM) is a central nervous system disorder and a type of central sensitivity syndrome [1]. The neurophysiologic basis of pain processing was recently evaluated with greater resolution using functional neuroimaging. Mountz et al., using single-photon emission computed tomography, observed bilateral hypoperfusion in the thalamus and caudate nucleus in women with fibromyalgia [1]. Gracely et al., using functional magnetic resonance imaging (MRI), revealed a significant relative increase in signal intensity in multiple pain-related brain regions in patients with FM after challenging both FM patients and controls with the same painful stimulus [2]. Based on a perfusion MRI study, Foerster et al. reported baseline changes in brain perfusion in patients with FM, especially in the thalami [3,4].

Garcia-Campayo et al. suggested that neuroimaging findings could be used to identify subgroups of FM patients, which would allow for personalized patient treatment [5]. These functional imaging techniques are expensive, however, and not readily available in a typical clinical practice. Currently, there are no easy and quick objective examinations for diagnosing FM or for assessing the severity of the disorder [6].

Visual loss is a main cause of disability in patients with neurodegenerative disease, and axonal loss in the retinal nerve fiber layer (RNFL) correlates with the extent of functional disability in patients with neurodegenerative disease, such as multiple sclerosis [7–10], Parkinson disease [9,11,12], and Alzheimer disease [13,14]. The RNFL comprises retinal ganglion cell axons that send information from the retina to the lateral geniculate nucleus. RNFL axons within the eye have no myelin sheath. In addition to being the main retinal component near the optic nerve (90% of retinal thickness), ganglion cells and their axons are also present in the macula (30%-35%) and can be quantified using noninvasive, rapid, objective, and reproducible ocular imaging technologies, such as optical coherence tomography (OCT), which is commonly used to evaluate the RNFL [13,15,16].

The aim of this study was to evaluate whether FM causes RNFL thinning and which subtypes or phases of this pathology exhibit high axonal damage in the optic nerve. We used two commonly available OCT devices to test which protocols more accurately detect whether FM causes axonal damage in the RNFL. Possible correlations of RNFL measurements in FM patients with disease duration, FM subgroup, functional ability, and quality of life were also analyzed.

## Materials and Methods

This is a prospective cohort study aiming to compare patients with FM (n = 119) against age- and sex-matched controls (n = 144).

Based on our previous studies, 86 subjects were needed to be able to detect significant differences in the RNFL thickness, assuming an alpha error of 5% and a beta error of 10%. A standard sample size equation was used to calculate the number of subjects required. To further increase the power of the study, we included 119 FM patients and 144 controls.

Patients were recruited from the primary care research group study population of FM patients in Zaragoza, Spain. This research group periodically evaluates all subjects with FM diagnosis or clinical suspects among healthy individuals of Zaragoza, which has a population of 850.000.

All participants signed their written informed consent to participate in this study. The written informed consents for patients and controls and the study protocol were approved by the Ethic Committee of Clinic Research in Aragon (CEICA) and by the Ethic Committee of Miguel Servet University Hospital, in Zaragoza, Spain. This study was conducted in accordance with the guidelines established by the principles of the Declaration of Helsinki.

The inclusion criteria were: confirmed FM diagnosis according to the 1990 American College of Rheumatology criteria for FM [17]; best corrected visual acuity ≥0.1 using a Snellen chart to allow the patients to complete the protocol; and intraocular pressure less than 21 mmHg to exclude other processes, such as open angle chronic glaucoma, related to RNFL thinning [18]. The exclusion criteria were: history of refractive errors (more than 5 diopters of spherical equivalent refraction or 3 diopters of astigmatism); concomitant ocular disease (such as glaucoma or retinal pathology); systemic pathologies that could impair the visual system; ocular trauma, laser therapy; and pathology affecting the optic nerve, cornea, retina, or lens (such as glaucoma, optic neuritis, keratoconus, macular degeneration, and cataract). Controls presented with no evidence of any disease. All procedures were performed in adherence to the tenets of the Declaration of Helsinki, and the local ethics committee approved the experimental protocol. Informed consent to participate in the study was provided by all subjects.

The FM patients were evaluated by a psychiatrist specializing in FM (blind to the ophthalmology evaluation), who registered measurements of disease duration since diagnosis; evaluation of FM severity using the Fibromyalgia Impact Questionnaire (FIQ); evaluation of activities of daily living and impact on quality of life using the Euro Quality of Life 5D (EQ-5D) scale.

All subjects were evaluated by two neuro-ophthalmologists (blind to the psychiatrist evaluation), who examined the anterior segment, assessed the best-corrected visual acuity based on the Snellen scale, and performed a visual field test, three different OCT protocols, and a fundoscopic exam. Each eye was considered independently and we randomly analyzed only one eye of each subject unless only one of the eyes met the exclusion criteria. We assessed the visual field using a Humphrey Field Analyzer (Carl-Zeiss Meditec, Dublin, CA). Mean deviation (dB), defect pattern, and pattern standard deviation were measured using the Swedish Interactive Threshold Algorithm Standard strategy (program 30–2).

### OCT evaluation

Peripapillary RNFL thickness measurements were obtained in all subjects using the Cirrus and Spectralis OCT devices. The OCT devices were used in random order to prevent fatigue bias. The same experienced operator performed all of the scans and was blinded to the results of the psychiatric and other ophthalmologic tests. A time delay was inserted between scan acquisitions and subject position and focus was randomly disrupted for adjustment of the alignment parameters at the beginning of each scan. The OCT output was not manually corrected. We used an internal fixation target to provide the highest level of reproducibility [19], and only analyzed images with a quality a score of >7 for the Cirrus OCT and >25 for Spectralis OCT. Three patients were excluded because poor fixation prevented us from acquiring a centered scan, and therefore only 116 patients were included in the statistical analysis. We also excluded 10 images due to artifacts, missing parts, or apparently distorted anatomy. Ten eyes were rescanned (six with the Cirrus OCT and four using the Spectralis OCT) to obtain good-quality and centered images [20].

**Fig 1:**
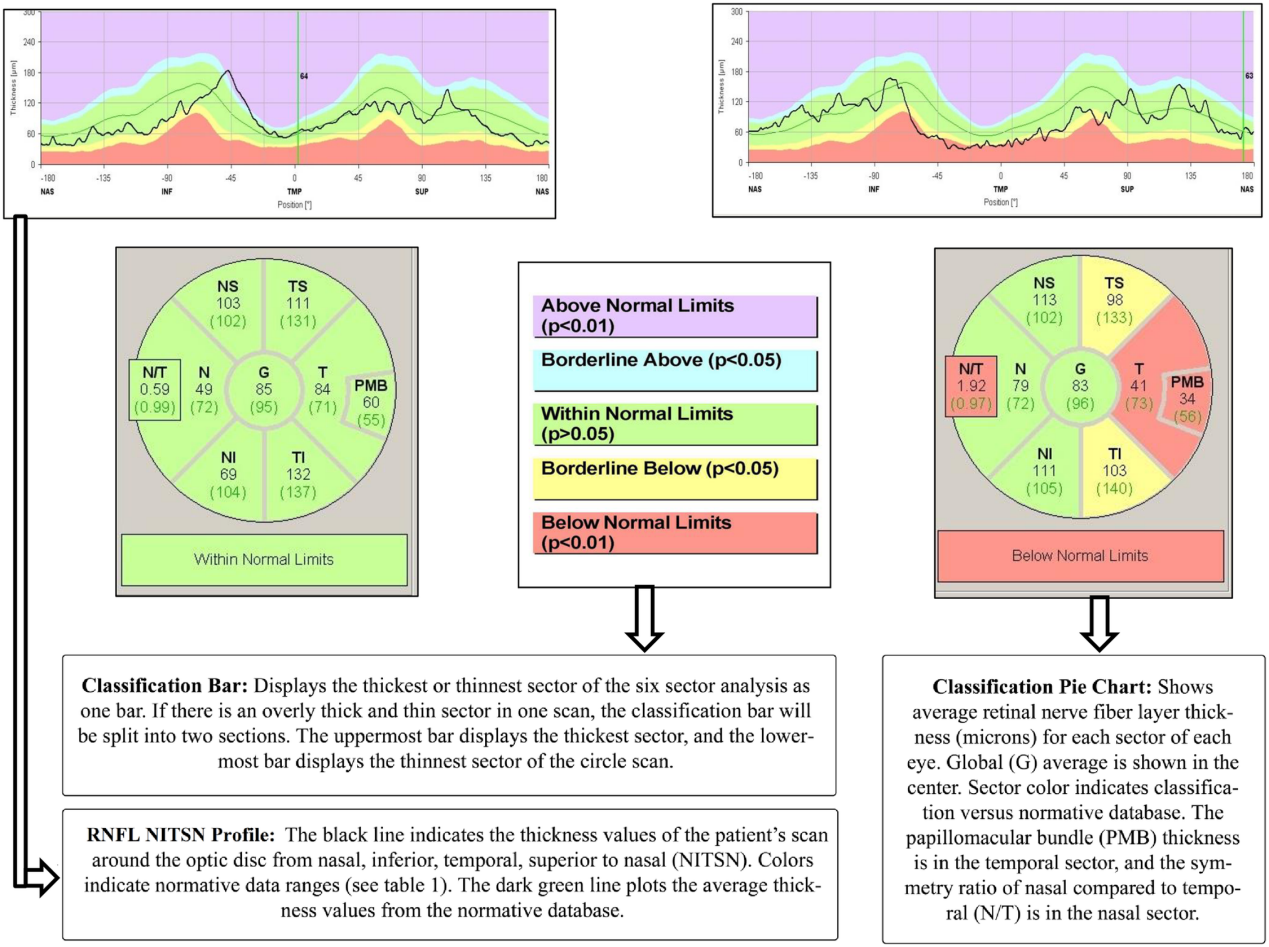
Representation and interpretation of the report from the Axonal application of the Spectralis optical coherence tomography in a control (left side) and in a patient with fibromyalgia and retina nerve fiber layer thinning (right side).

The Cirrus Macular Cube 200 x 200 protocol was performed using the Cirrus OCT. Each scan was analyzed using the ganglion cell layer analysis application to measure the mean and minimum inner plexiform, the fovea, and the nasal and temporal sectors. With the Spectralis OCT, we used two different RNFL applications in each of the subjects: the classic Glaucoma application and the Axonal application (created specifically to evaluate neurologic diseases that impact the RNFL). The images were acquired using TruTrack eye-tracking technology. Both RNFL applications generate a thickness map indicating mean overall thickness, mean thickness for each of the superior, inferior, nasal and temporal quadrants, and mean thickness of the nasal superior, nasal, nasal inferior, temporal inferior, temporal, and temporal superior sectors (Fig 1). RNFL defects were identified by comparing each patient’s measurements with those of the normative database provided with each instrument. The Axonal application utilizes fovea-to-disc technology to orient the anatomy correctly and to minimize variability due to differences in patient head orientation. In addition, the Axonal system places the temporal region of the scan in the center of the viewing window to enhance analysis of papillomacular bundle axonal loss, which occurs in neurodegenerative disease. The RNFL thickness graph displays the scan results in the quadrants, and provides two additional neuro-ophthalmologic parameters: papillomacular bundle thickness and nasal/temporal (N/T) ratio.

### Assessment of the Fibromyalgia syndrome

Disease duration since diagnosis was recorded (in months) and all patients were classified following FM subgrouping at the Miguel Servet Hospital Fibromyalgia Unit, based on the pressure-pain thresholds and psychologic factors described by Giesecke et al [21]. Despite the existence of several FM subgroup classifications, Giesecke’s classification method is considered highly reliable by FM researchers and specialists because it is based on both psychologic and biologic measures. The Giesecke classification includes three subgroups: Subgroup 1 (atypical): low tenderness, moderate depression/anxiety, moderate catastrophizing, and moderate control over pain; Subgroup 2 (depressive): high tenderness, high depression/anxiety, high catastrophizing, and low control over pain; and Subgroup 3 (biologic): high tenderness, low depression/anxiety, low catastrophizing, and high control over pain. The questionnaires and examinations used to classify FM patients into these subgroups are described in Giesecke et al. [21].

We used the FIQ because it has reliable test-retest characteristics, credible construct validity, and good sensitivity for demonstrating therapeutic effects in patients with FM [22]. Patients in the present study completed the FIQ and were assigned a score of 0 through 100. The higher the score, the greater the disease impact. We used the validated Spanish version of the FIQ [23].

We assessed the symptoms of patients with FM and the impact on activities of daily living using the Euro-quality of life questionnaire (EQ-5D). It is critical to assess health-related quality of life outcomes in FM patients because of the multitude of symptoms, which include chronic pain, fatigue, weakness, hyperalgesia, secondary depression, and allodynia. These symptoms can substantially impair the patients’ ability to work and disrupt their quality of life by interfering with social and family functioning. The EQ-5D comprises five questions with three response categories involving the following dimensions: mobility, self-care, usual activities, pain, and anxiety/ depression. The EQ-5D results are expressed as the percentage of subjects with moderate or major problems [24]. We used the validated Spanish version of the EQ-5D [25].

### Statistical analysis

Data were stored in a custom database (File Maker Pro, version 8.5) The Statistical Package for the Social Sciences (SPSS 20.0, SPSS Inc., Chicago, IL) was used for the statistical analyses. Sample distribution was assessed using the Kolmogorov-Smirnov test, and therefore mean, standard deviation and standard error of the mean were used in the descriptive analysis, and parametric tests were used for comparison.

The OCT and ophthalmologic parameters were normally distributed and differences between controls and FM patients were assessed using unpaired Student’s t-test (two sided) and analysis of variance (ANOVA). Bonferroni’s correction for multiple comparisons was used to adjust for multiple comparisons (p-value with Bonferroni´s correction was 0.012). The odds ratio (OR) was calculated to estimate the size effect of FM in RNFL thinning.

The FM population was divided in two subgroups: 68 patients with severe FM (FIQ≥60) and 48 patients with mild FM (FIQ<60). We compared both subgroups with controls to evaluate the presence of RNFL atrophy in even the early stages of FM disease. We also evaluted the differences among the biologic FM, depressive FM, and atypical FM subgroups. Correlations between OCT measurements, disease duration, severity (measured by FIQ), and impact on quality of life (by EQ-5D) were calculated using Pearson’s correlation coefficient. We analyzed which OCT measurements showed better sensitivity for detecting disease severity (FIQ) and impact on quality of life (EQ05D) in FM patients, using a multivariate logistical regression analysis.

## Results

We evaluated a total of 116 patients with FM and 144 age- and sex-matched controls. Mean age was 52.13±8.34 years in the FM group and 51.11±8.98 years in the control group. Age, sex, and intraocular pressure did not differ significantly between FM and control groups.

Table 1 shows the epidemiologic and disease characteristics. FM duration ranged from 6 months to 24 years with a median of 7.9 years since diagnosis. The FM phenotype distribution was: biologic FM, 26 patients (22.4%); depressive FM, 34 patients (29.3); atypical FM, 56 patients (48.3%). The FIQ scores ranged from 18.39 to 97.61 (mean 62.15). The EQ-5D scores ranged from 5 to 95 (mean 45.05).

**Table 1:** Epidemiologic and disease characteristics of 116 patients with fibromyalgia syndrome and 144 controls included in the study, and statistical significance of the comparisons between the groups (P).

	Patients with Fibromyalgia syndrome (n = 116)	Controls (n = 144)	P*
Age (years): mean (range)	52.1 (33–69)	51.1 (32–70)	0.393
Men:Women (% women)	4:112 (98.2%)	6:138 (95.83%)	0.103
Disease duration (years): mean (SD)	7.88 (4.23)	-	-
FIQ: mean (SD)	62.1 (18.72)	-	-
EQ-5D: mean (SD)	45.0 (18.84)	-	-
Intraocular pressure (SD)	14.1 (1.21)	14.0 (1.32)	0.540
BCVA (ETDRS scale): mean (SD)	-0.01 (0.46)	-0.02 (0.35)	0.607
MD Visual Field [mean (SD)]	1.9 (3.14)	-0.4 (2.22)	0.212
PSD Visual Field (dB) [mean (SD)]	1.2 (1.74)	1.34 (1.04)	0.324

Abbreviations: EQ-5D, FIQ, Fibromyalgia Impact Questionnaire; European Quality of Life-5 Dimensions; BCVA, best corrected visual acuity; ETDRS, Early Treatment Diabetic Retinopathy Study; dB, decibels; MD, mean deviation of visual field; PSD, pattern standard deviation of visual field;.

*Significant difference (P<0.05) between normal subjects and Fibromyalgia syndrome patients for each population were calculated using unpaired Student’s t-test (two sided).

Best corrected visual acuity, mean deviation, and standard deviation of the visual field did not differ significantly between FM patients and controls (Table 1) (S1 Table. Minimal datset)

### RNFL thickness comparisons between FM and controls

**Fig 2:**
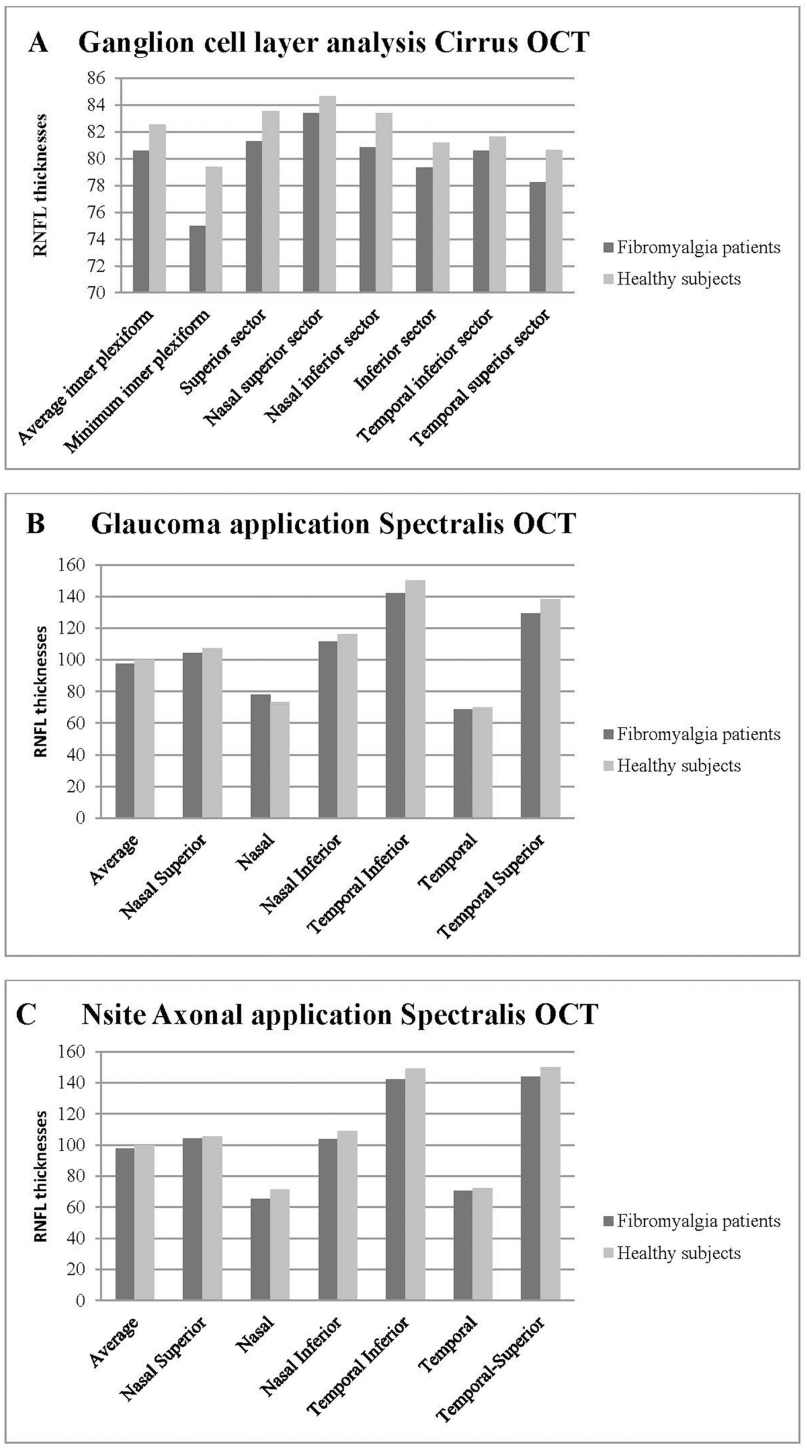
Bar charts representing optical coherence tomography (OCT) measurements in eyes from 116 fibromyalgia patients and 144 controls. A- Retinal nerve fiber layer measurements using the ganglion cell layer analysis of Cirrus OCT device. B- Retinal nerve fiber layer measurements using the Glaucoma analytic application of the Spectralis OCT device. C- Retinal nerve fiber layer measurements using the Nsite RNFL Axonal application of the Spectralis OCT device.

Based on findings from both OCT devices, FM patients had significantly reduced RNFL thickness (Table 2, Fig 2). The Cirrus OCT ganglion cell layer analysis revealed significant differences in the minimum inner plexiform, the nasal inferior, temporal inferior, and temporal superior sector thicknesses in FM patients (see Table 2).

**Table 2:** Mean and standard error of the mean of retinal nerve fiber layer thicknesses obtained with the Cirrus and Spectralis optical coherence tomography devices in patients with Fibromyalgia syndrome and controls, and statistical significance.

		Fibromyalgia patients (n:116)		Controls (n:144)		P
		Mean	SEM	Mean	SEM	
**Cirrus Ganglion cell layer analysis**	Average Inner Plexiform	80.6	1.02	82.5	0.85	0.207
	Minimum Inner Plexiform	75.0	0.99	79.4	0.72	**0.023**
	Fovea	257.4	1.67	258.3	1.41	0.728
	Superior sector	81.3	1.19	83.5	0.78	0.166
	Nasal Superior sector	83.4	1.17	84.6	1.03	0.535
	Nasal Inferior sector	80.8	1.54	83.4	1.06	**0.040**
	Inferior sector	79.3	1.22	81.2	0.68	0.300
	Temporal Inferior sector	78.6	1.39	81.6	0.98	0.011*
	Temporal Superior sector	77.2	0.97	80.7	1.01	**0.046**
**Spectralis RNFL(Glaucoma application)**	Average	97.6	1.02	99.9	0.97	0.114
	Nasal Superior sector	104.3	2.73	107.2	2.72	0.376
	Nasal sector	73.0	1.96	78.5	1.17	0.009*
	Nasal Inferior sector	111.7	2.98	116.1	2.73	0.277
	Temporal Inferior sector	142.1	2.24	150.2	1.81	0.006*
	Temporal sector	68.8	1.59	70.1	0.96	0.498
	Temporal Superior sector	129.5	2.28	138.1	1.53	0.002*
**Spectralis RNFL-N(Axonal application)**	Average	97.5	1.12	100.0	1.01	**0.047**
	Nasal Superior sector	104.1	1.32	105.4	1.21	0.123
	Nasal sector	65.4	1.21	71.3	1.34	0.006*
	Nasal Inferior sector	103.6	1.60	108.9	1.55	0.002*
	Temporal Inferior sector	142.1	0.83	149.0	0.79	<0.001*
	Temporal sector	70.4	0.95	72.4	0.88	0.187
	Temporal Superior sector	143.9	1.32	150.0	1.21	<0.001*
	Papillomacular buddle	53.6	0.80	57.5	0.77	<0.001*
	Nasal/Temporal index	0.89	0.01	1.01	0.01	0.012*

Thickness measurements are in microns (μm). Abbreviations: SEM, standard error of the mean; RNFL, retinal nerve fiber layer. P: Student’s t-test between normal and Fibromyalgia disease groups.

Numbers in bold indicate significant difference (P<0.05)

* indicate significant difference using Bonferroni correction for multiple comparisons for each application of optical coherence tomography device (p<0.012).

The odds ratio (OR) was calculated to estimate the size effect of FM in RNFL thinning using the following formula: OR = (a*d) / (b*c). The coefficient “a” indicates FM patients who present with a thickness reduction in at least one temporal sector or the papillomacular buddle of the Axonal application of the Spectralis OCT (using the normative database of this device). The coefficient “b” indicates healthy subjects with a thickness decrease in those parameters. The coefficients “c” and “d” indicate FM patients and healthy controls without a thickness decrease in those parameters, respectively. The OR obtained was 1.39.

The two applications used with the Spectralis OCT (the classic Glaucoma application RNFL protocol and the Axonal application RNFL-N protocol) revealed significant differences in the nasal and temporal sectors in FM patients. The Axonal application also revealed that FM patients had a significant thinning of the papillomacular bundle and a significant increase in the N/T (nasal/temporal) index (Table 2; Fig 1). Although other sectors showed clear tendencies toward RNFL thinning (Fig 2), the differences were not statistically significant (Table 2).

### Subgroup analysis

Based on the FIQ, the FM population was divided in two subgroups: 68 patients with severe FM (FIQ≥60) and 48 patients with mild FM (FIQ<60). Both subgroups exhibited statistically significant differences when compared with controls in Temporal Inferior sector using Cirrus Ganglion cell layer analysis, in Temporal Superior and Temporal Inferior sectors using Glaucoma RNFL application of Spectralis OCT and in all measurements provided by Axonal RNFL-N application of Spectralis OCT (except Average thickness, Nasal Superior and Temporal sectors) (Table 3). The RNFL temporal inferior sector thickness obtained with both Spectralis OCT applications was significantly reduced in patients with severe FM (FIQ≥60) compared with patients with mild FM (FIQ<60; p = 0.032 with the Glaucoma application and 0.021 with Axonal application). Based on Bonferroni´s correction, the differences between severe and mild FM were not statistically significant. Based on the Axonal application, RNFL temporal superior thickness in patients with severe FM was significantly reduced compared with patients with an FIQ score lower than 60 (p = 0.018).

**Table 3:** Mean and standard error of the mean of retinal nerve fiber layer thicknesses obtained with the Cirrus and Spectralis optical coherence tomography devices in patients with early and advantage stages of Fibromyalgia syndrome and statistical significance with controls.

		Fibromyalgia patients with FIQ<60 (n:48)			Fibromyalgia patients with FIQ≥60 (n:68)		
		Mean	SEM	P*	Mean	SEM	P**
**Cirrus Ganglion cell layer analysis**	Average Inner Plexiform	81.0	1.11	0.433	80.3	0.98	0.329
	Minimum Inner Plexiform	76.0	0.78	0.102	74.1	1.23	**0.018**
	Fovea	258.2	1.64	0.435	256.7	1.71	0.691
	Superior sector	81.8	1.05	0.237	81.1	1.34	0.430
	Nasal Superior sector	83.6	1.16	0.349	83.2	1.18	0.667
	Nasal Inferior sector	82.0	1.44	0.056	81.2	1.50	**0.031**
	Inferior sector	78.9	1.15	0.543	79.1	1.30	0.211
	Temporal Inferior sector	78.7	1.46	**0.020**	78.3	1.32	**0.038**
	Temporal Superior sector	78.2	1.02	**0.039**	77.8	0.95	0.067
**Spectralis RNFL (Glaucoma application)**	Average	98.1	0.91	0.609	97.8	1.13	0.078
	Nasal Superior sector	106.8	2.57	0.441	103.0	3.01	0.601
	Nasal sector	75.9	1.88	0.098	69.6	2.10	**0.022**
	Nasal Inferior sector	113.3	3.13	0.654	109.5	2.67	0.265
	Temporal Inferior sector	145.8	2.23	0.012*	139.0	2.26	0.003*
	Temporal sector	67.0	1.47	0.342	68.3	1.78	0.561
	Temporal Superior sector	131.5	2.29	0.002*	127.3	2.26	0.009*
**Spectralis RNFL-N (Axonal application)**	Average	98.7	1.10	0.078	97.8	1.08	**0.038**
	Nasal Superior sector	104.3	1.43	0.345	104.9	1.21	0.098
	Nasal sector	67.3	1.26	0.004*	64.9	1.16	0.007*
	Nasal Inferior sector	105.8	1.55	0.003*	102.6	1.66	0.002*
	Temporal Inferior sector	145.4	0.80	0.001*	139.8	0.87	<0.001*
	Temporal sector	71.9	0.80	0.453	71.0	1.34	0.089
	Temporal Superior sector	146.4	1.23	<0.001*	141.9	1.42	<0.001*
	Papillomacular buddle	55.9	0.83	0.003*	53.6	0.78	<0.001*
	Nasal/Temporal index	0.98	0.01	0.010*	0.98	0.01	0.008*

Thickness measurements are in microns (μm). Abbreviations: FIQ, Fibromyalgia Impact Questionnaire; SEM, standard error of the mean; RNFL, retinal nerve fiber layer.

P*: Student’s t-test between controls and Fibromyalgia patients with smaller disease severity (FIQ<60).

P**: Student’s t-test between controls and Fibromyalgia patients with higher disease severity (FIQ≥60). Numbers in bold indicate significant difference (P<0.05).

Numbers in bold indicate a significant difference (P<0.05)

* indicates a significant difference using the Bonferroni correction for multiple comparisons for each application of the optical coherence tomography device (p<0.012).

The FM population was also divided in three subgroups depending on Giesecke’s classification of FM using the same instruments: biologic FM (26 patients), depressive FM (34 patients), and atypical FM (56 patients). Statistical differences were detected between patients with biologic FM and the other two groups in the thicknesses of the temporal inferior and temporal superior sectors using the Spectralis OCT Axonal application: the biologic FM subgroup had significant thinning in the temporal inferior and superior sectors (115.17±20.82 μm and 117.05±24.19 μm, respectively) compared with the depressive FM (130.83±22.97 μm and 127.71±26.10 μm, respectively) and atypical FM (128.60±26.54 μm and 125.55±23.65 μm, respectively) subgroups (ANOVA; p = 0.005 for the temporal superior sector and p = 0.001 for the temporal inferior sector) (Table 4).

**Table 4:** Mean and standard error of the mean of retinal nerve fiber layer thicknesses obtained with the Cirrus and Spectralis optical coherence tomography devices in patients with biologic fibromyalgia (26 patients), depressive fibromyalgia (34 patients), and atypical fibromyalgia (56 patients).

		Biologic fibromyalgia		Depressive fibromyalgia		Atypical fibromyalgia		P
		Mean	SEM	Mean	SEM	Mean	SEM	
**Cirrus Ganglion cell layer analysis**	Average Inner Plexiform	80.2	0.88	77.5	1.21	81.7	0.95	0.452
	Fovea	258.4	0.97	254.9	1.10	258.1	1.00	0.391
	Inferior sector	78.5	1.55	75.5	1.78	81.0	1.67	0.552
	Minimum Inner Plexiform	73.9	1.03	72.7	0.98	75.4	1.28	0.418
	Nasal Inferior sector	81.3	1.22	75.9	1.19	82.6	1.02	0.691
	Nasal Superior sector	82.7	1.50	79.5	1.59	85.2	1.57	0.379
	Superior sector	80.5	1.31	79.7	1.33	81.7	0.99	0.672
	Temporal Inferior sector	79.8	1.41	77.3	1.35	82.0	1.27	0.590
	Temporal Superior sector	78.8	0.94	76.4	0.97	78.2	1.01	0.872
**Spectralis RNFL(Glaucoma application)**	Average	96.2	1.23	94.1	1.11	98.6	0.92	0.315
	Nasal Superior sector	103.3	2.77	103.4	2.80	105.2	2.65	0.470
	Nasal sector	82.8	2.08	75.5	1.77	77.1	2.11	0.102
	Nasal Inferior sector	115.2	3.16	104.0	2.65	109.6	3.03	0.247
	Temporal Inferior sector	142.1	2.31	130.8	2.03	146.3	2.21	0.111
	Temporal sector	63.9	1.46	67.9	1.55	69.8	1.34	0.199
	Temporal Superior sector	117.0	1.80	127.7	1.96	133.7	2.09	0.367
**Spectralis RNFL-N(Axonal application)**	Average	97.0	1.23	97.8	1.13	99.1	1.01	0.890
	Nasal Superior sector	102.1	1.32	104.7	1.31	103.8	1.34	0.498
	Nasal sector	66.7	1.01	67.9	1.33	68.1	1.28	0.550
	Nasal Inferior sector	105.2	1.60	106.0	1.70	106.2	1.50	0.669
	Temporal Inferior sector	115.2	0.80	130.8	0.84	128.6	0.85	**0.005**
	Temporal sector	70.8	1.15	72.4	1.04	72.4	0.88	0.209
	Temporal Superior sector	117.0	1.27	127.7	1.35	125.5	1.31	**0.001**
	Papillomacular buddle	53.5	0.78	56.0	0.83	54.6	0.78	0.255
	Nasal/Temporal index	1.02	0.01	0.98	0.01	0.98	0.01	0.098

Thickness measurements are in microns (μm). Abbreviations: SEM, standard error of the mean; RNFL, retinal nerve fiber layer.

### Correlation analysis and logistic regression between OCT and disease parameters

Measurements of the RNFL by the Cirrus OCT and by the Spectralis OCT’s Glaucoma application were significantly positively correlated (r = 0.789, p<0.001 between the mean inner plexiform layer thickness of the Cirrus OCT and mean RNFL thickness of the Glaucoma application). Similarly, RNFL measurements by the Cirrus OCT and the Spectralis OCT’s Axonal application were significantly positively correlated (r = 0.767; p<0.001, between the mean inner plexiform of the Cirrus, and the RNFL mean thickness of the Axonal application). Correlation between the two Spectralis applications was good (r = 0.881; p<0.001, RNFL mean thickness). Mean RNFL thickness values obtained with the three applications differed significantly (ANOVA, p = 0.016). The RNFL thicknesses provided by the three applications did not significantly correlate with disease duration or with FIQ and EQ-5D scores. Based on stepwise logistic regression analysis (using step-forward selection), the OCT parameters did not predict disease severity in FM patients (low FIQ score) or impaired quality of life (low EQ-5D score). Although we detected a correlation between OCT parameters, FIQ, and EQ-5D scores, this association did not demonstrate prognostic utility in this study.

## Discussion

The main finding in the present study evaluating RNFL parameters in patients with FM was the presence of axonal damage in the optic nerve of FM patients, even in early stages of the disease. Our results revealed that even patients with mild FM (FIQ<60) exhibited subclinical RNFL atrophy in the temporal sectors.

Currently, FM lacks a specific and definitive diagnostic test. The results of the present study provide a new option to facilitate the diagnosis of FM. The ability to evaluate the optic nerve as an indicator of the disease is an important advance, and this examination can be easily implemented in clinical practice, because OCT tests are noninvasive, fast, and comfortable for patients, as well as inexpensive.

Previous studies described neurobiologic and structural brain abnormalities in FM [26,27]. Clauw described FM as a "central sensitization syndrome" caused by neurobiologic abnormalities that produce neuropsychologic symptoms [27]. Our findings support this emerging theory and contribute new knowledge regarding the etiology of this little-known disease because we observed axonal damage in the optic nerve, which suggests that neurodegeneration contributes to the pathology of FM.

The ophthalmologic tests described in this study allow for the eye to be utilized as a “window” to the central nervous system, specifically to directly observe the axons in the optic nerve. Previous studies demonstrated that eye assessment can provide useful information for early diagnosis of some neurodegenerative diseases, such as Parkinson disease, multiple sclerosis, and Alzheimer disease [8,9,13,16]. The optic nerve is a cranial nerve considered part of the central nervous system because it derives from the diencephalon during embryonic development. Once the fibers exit the eye globe, they are covered with myelin produced by oligodendrocytes, which are myelinating cells in the central nervous system. Peripheral neuropathies, such as Guillain-Barré syndrome, do not affect the optic nerve.

The first portion of the optic nerve is one millimeter long and locates within the eyeball. This portion of the optic nerve lacks myelin or meningeal layers, and thus axonal damage can be evaluated in this segment using OCT and high resolution photographs. OCT is suggested to be more accurate than MRI for quantifying axonal damage in multiple sclerosis patients [28–30]. RNFL thickness is well-correlated with MRI measurements of the brain, such as the brain volumes and the parenchymal fraction, and mean RNFL thickness is strongly associated with normalized brain volume [29,31].

The fibers of the temporal quadrant follow the papillomacular bundle, so the temporal RNFL quadrant is most often affected in early neurodegenerative diseases [32]. Our results in FM patients are consistent with these findings: we have found that papillomacular bundle thickness decreased in patients with FM with the temporal sector being the most vulnerable (the N/T index was higher in the FM group because the temporal quadrant was affected more by the RNFL thinning than the other sectors).

We also detected differences between FM subgroups. Patients with biologic FM (low depression/anxiety/catastrophizing but high tenderness) had a significant decrease in the temporal inferior and temporal superior sectors compared with patients suffering from depressive or atypical FM, suggesting the presence of neurodegenerative processes in the biologic FM subgroup. These findings are reasonable because these patients have a high pain level but not the associated psychiatric disorders (depression/anxiety) that explain this pain, so a lesion of the tissues would be the most sensible explanation. Studies with a larger sample size will likely detect greater differences between the three FM subgroups.

OCT evaluation is noninvasive, inexpensive, fast, and comfortable for the patient. OCT does not require pupil dilation, and thus does not impact the patient’s vision. While patients may occasionally be temporarily blinded following the test, this lasts only 1 or 2 minutes. Obviously, FM patients should be diagnosed and followed-up by a rheumatologist, but ophthalmologic tests are new, noninvasive, and cost-effective tools than can be used to facilitate the diagnosis of FM and may reduce the expenses associated with diagnosis of this disease. Evaluation of the RNFL in FM patients will enhance our knowledge of the disease etiology.

The two applications used with the Spectralis OCT (the classic Glaucoma application RNFL protocol and the Axonal application RNFL-N protocol) revealed significant differences in the nasal and temporal sectors in FM patients. The Axonal application also revealed that FM patients had a significant thinning of the papillomacular bundle and a significant increase in the N/T (nasal/temporal) index (Table 2; Fig 1). Although other sectors showed clear tendencies toward RNFL thinning (Fig 2), the differences were not statistically significant (Table 2).

This study has some limitations. First, although we checked that both groups (patients and controls) had no differences in refractive error, we did not evaluate axial length, which may interfere with the results. Second, our logistic regression analysis found that OCT parameters did not predict disease severity in FM patients, although we did detect correlations. Studies with a larger sample size or longitudinal studies may find a predictive association between RNFL thinning and FM severity or changes in the quality of life. Third, in this study, we used no internal or external validation techniques to corroborate the results. Finally, we found that the RNFL temporal inferior sector thickness obtained with both Spectralis OCT applications was significantly reduced in patients with severe FM (FIQ≥60), but these results were not statistically significant when Bonferroni´s correction was applied, so this difference is uncertain. Again, studies with a larger sample size might shed more light on this point.

Longer-term studies are required to evaluate the clinical application of RNFL measurements in FM patients as a diagnostic tool, to follow disease progression, to identify patients with worse prognosis or at higher risk for loss of quality of life, and to measure treatment effectiveness.

## Supporting Information

S1 Table Minimal Datset that shows results of visual function parameters in our population. Abbreviations: ETDRS, Early Treatment Diabetic Retinopathy Study; OCT, Optical Coherence Tomography; GCL, Ganglion Cell Layer; IPL, Inner Plexiform Layer; CSV, Contrast Sensitivity Vision; CVR, Color Vision Recorder; AC CCI, age corrected color confusion index; CCI, color confusion index; C-index, confusion index; Conf Angle, confusion angle; S-index, scatter index.
(DOCX)
