# Supplementary material for: Fibromyalgia Is Correlated with Retinal Nerve Fiber Layer Thinning
Source: PLoS One. 2016 Sep 1;11(9):e0161574. doi: 10.1371/journal.pone.0161574 (PMC5008644; doi:10.1371/journal.pone.0161574)
Supplement: S1 Table — Abbreviations: ETDRS, Early Treatment Diabetic Retinopathy Study; OCT, Optical Coherence Tomography; GCL, Ganglion Cell Layer; IPL, Inner Plexiform Layer; CSV, Contrast Sensitivity Vision; CVR, Color Vision Recorder; AC CCI, age corrected color confusion index; CCI, color confusion index; C-index, confusion index; Conf Angle, confusion angle; S-index, scatter index. (DOCX) [file pone.0161574.s001.docx]

|  | Range | Minimum | Maximum | Mean | Standard Deviation | Variance |
| --- | --- | --- | --- | --- | --- | --- |
| ETDRS 100% | 4.92 | -4.00 | 0.92 | -0.0121 | 0.46512 | 0.216 |
| ETDRS 2.5% | 1.08 | -.008 | 1.00 | 0.3182 | 0.15472 | 0.024 |
| Pelli Robson | 1.95 | .000 | 1.95 | 1.8213 | 0.25888 | 0.067 |
| Stereopsis test | 480 | 0 | 480 | 117.24 | 114.266 | 13056.672 |
| OCT Average GCL IPL | 96 | 23 | 119 | 80.76 | 11.235 | 126.234 |
| OCT Fovea GCL | 62.96 | 222.65 | 285.61 | 257.473 | 7.4698 | 55.798 |
| OCT Minimun GCL IPL | 91 | 7 | 98 | 75.39 | 15.933 | 253.856 |
| OCT Superior GCL | 88 | 16 | 104 | 81.49 | 11.822 | 139.752 |
| OCT Nasal GCL | 159 | 20 | 179 | 83.50 | 14.855 | 220.685 |
| OCT Inferior GCL | 165 | 14 | 179 | 79.49 | 15.683 | 245.969 |
| OCT Temporal GCL | 77 | 23 | 100 | 78.46 | 11.091 | 123.017 |
| CSV1000 Frequency A | 0.74 | 1.34 | 2.08 | 1.7030 | 0.16661 | 0.028 |
| CSV1000 Frequency B | 0.91 | 1.38 | 2.29 | 1.8991 | 0.18008 | 0.032 |
| CSV1000 Frequency C | 1.38 | 0.61 | 1.99 | 1.5097 | 0.28336 | 0.080 |
| CSV1000 Frequency D | 1.38 | 0.17 | 1.55 | 1.0266 | 0.28190 | 0.079 |
| CVR Farnsworth AC CCI | 1.80 | 0.15 | 1.95 | .9678 | 0.14184 | 0.020 |
| CVR Farnsworth C- index | 0.62 | 1.00 | 1.62 | 1.0360 | 0.10159 | 0.010 |
| CVR Farnsworth CCI | 0.54 | 1.00 | 1.54 | 1.0263 | 0.07584 | 0.006 |
| CVR Farnsworth Conf Angle | 45.30 | 41.50 | 86.80 | 62.4027 | 4.37225 | 19.117 |
| CVR Farnsworth S-index | 1.14 | 1.20 | 2.34 | 1.5178 | 0.13418 | 0.018 |
| CVR L´ Anthony AC CCI | 1.10 | 0.77 | 1.87 | 0.9933 | 0.1877 | 0.035 |
| CVR L´ Anthony C- index | 2.34 | 1.00 | 3.34 | 1.2898 | 0.3696 | 0.137 |
| CVR L´ Anthony CCI | 139.00 | 1.00 | 140.00 | 2.4346 | 13.1178 | 172.077 |
| CVR L´ Anthony Conf Angle | 171.8 | -83.9 | 87.9 | 62.306 | 21.2496 | 451.547 |
| CVR L´ Anthony S-index | 1.82 | 1.23 | 3.05 | 1.6955 | 0.36710 | 0.135 |
